# The impact of emotional labor on the health in the workplace: a narrative review of literature from 2013–2018

**DOI:** 10.3934/publichealth.2019.3.268

**Published:** 2019-08-20

**Authors:** Norah Aung, Promise Tewogbola

**Affiliations:** Department of Health Science and Social Work, Western Illinois University, 1, University Circle, Macomb, Illinois, USA

**Keywords:** emotional labor, stress, workplace, fatigue, workplace stress

## Abstract

There is a paradigm-shift in the workplace from a production mentality to a service-oriented mentality. As a result, there is a greater need on employees to expend emotional labor in dealing with the challenges of meeting the demands of a service-oriented economy. This present study discussed the impact that expending emotional labor has on the health of employees in the workplace. Literature was retrieved from MEDLINE/PubMed, Excerpta Medica/EMBASE, Scopus and Thomson Reuters' Web of Science databases. Studies were selected if they were published between 2013 and 2018, written in English and had the terms “emotional labor” or “emotional labour” in their titles. An overview of the different effects of emotional labor on the health of employees in different sectors of the economy revealed effects ranging from burnout and fatigue to dysmenorrhea, disruptions in sleep patterns and suicidal tendencies. The effects of emotional labor on the health of members of the population who belong to the working class can be attributed to reciprocal determinism where environmental influences in the form of clients, supervisors, or organizational culture put employees in a position where they had to develop the habit of engaging emotional labor to cope with environmental stresses.

## 1. Introduction

In today's modern society, there is an increase in the level of importance ascribed to the delivery of services as more economies are making the leap from a manufacturing-oriented economy to one that is service-oriented [1]. This increase has led to a surge in the number of service-based occupations to meet the rising demand [1]. However, coupled with the increased demand for service-based jobs, there is a corresponding increase in the exposure of these employees to varying degrees of stress and abuse, not excluding those of physical, psychological and emotional implications [1]. To cope with these stresses, employees with service-based occupations need to find a way to regulate or suppress their emotions in order to carry out their job responsibilities professionally [1]. The need to cope with abuse and stress in the work environment is not limited to service-based occupations, but among employees from diverse vocational fields [2].

Emotional labor can be described as the management of a person's feeling, which contributes to creating a facial and bodily display that is publicly observable [3], and can also impact an individual effectiveness on a job [4]. Employees use emotional labor to regulate and/or suppress their emotions during encounters with clients, colleagues, as well as superiors. Generally, emotional labor is characterized by three requirements: voice-to-voice contact with clients, an emotional state crucial for job duties, as well as, training and/or supervision from employers on how employees control their emotions [5]. For these reasons, the term ‘emotional labor’ is most appropriate when efforts expended in providing emotional exertion are ultimately rewarded with some kind of valued remuneration [2].

Furthermore, emotional labor can lead to health complications such as high blood pressure, exhaustion, diseases of the heart and emotional trauma [6]. Studies have shown that emotional labor may be associated with violence at workplace [7]. For instance, according to the Bureau of Labor Statistics (2018) [8], 500 homicides were recorded in 2015 with 27 percent of these deaths carried out by coworkers or work associate. This number may indicate the tipping point where continual emotional labor may spill over into outright acts of violence in the workplace. Emotional labor is also an important factor in sport especially in its leadership. Sport leadership roles require a lot of emotional balance in order to keep the team at optimal performance [9].

Studies have revealed that emotional labor is positively associated with an increase in the quality of service perceived by clients, especially since emotional labor can dramatically influence consumer loyalty, client satisfaction and financial outcomes [10]. Consequently, emotional labor is considered to be a requisite element of professional roles which is expected during the course of performing one's professional duties [11]. In fact, it is so important that in some professions, the inability to engage in emotional labor is the same as being totally incompetent in the job [12]. For instance, social workers are not expected to shed tears in the presence of their clients, while members of the police force, as well as emergency responders would be deemed as unprofessional if they display the emotion of fear or anxiety when faced with an emergency [12]. Studies have also reported that emotional labor exerts negative influence on job satisfaction, employees' well-being, emotional exhaustion, organizational commitment, intentions to leave, as well as employee turnover [10]. The purpose of this paper is to dive deeper by providing a narrative review of the impacts that emotional labor could specifically have on the health of employees in the workplace.

## 2. Search methodology

The journal articles considered for this narrative review were obtained by searching the National Library of Medicine's MEDLINE/PubMed database, as well as Excerpta Medica/EMBASE, Scopus and Thomson Reuters' Web of Science for open access research papers that were written on the topic of emotional labor. Only papers written in English between the years 2013 and 2018 were considered. The two search terms that were used in searching for papers that met the inclusion criteria include “emotional labor” and “emotional labour”. A total of 10 research papers were identified for inclusion in this review. The search of MEDLINE/PubMed, Excerpta Medica/EMBASE, Scopus and Thomson Reuters' Web of Science databases provided a total of 3289 citations. Of these, 3270 papers were discarded because after reviewing their abstracts, they appeared not to meet the criteria for the study. Two papers were removed because they were conceptual papers. A further two papers were removed because they were mere literature reviews based on emotional labor. One paper was removed because it was concerned solely with the development of a scale for measuring emotional labor. Finally, two more papers were removed because they were written in the Polish language, even though the abstract was written in English. Of the 10 research papers that were used in this narrative review, 8 studies were carried out in Asia, while the remaining 2 were carried out in Europe.

## 3. Discussion

This study shows the impact of emotional labor demonstrated in different professions. There is a trend of decrease in work efficiency due to emotional labor resulting into cases such as depression, dissonance, cardiovascular disease, exhaustion and suicidal tendency. The health conditions of a worker as a huge impact on the workforce affecting the result of their duties. Some of the professions this study revealed are as follows;

### 3.1. Impact of emotional labor on the health of teachers

In a study of 39 German secondary school teachers, the paramount emotion they felt was enjoyment and anger, although, they also reported feeling anxiety during lessons to a lesser extent [13]. The teachers reported expending a moderate amount of effort in engaging in emotional labor, although this emotional labor was spent in suppressing their paramount emotions, rather than faking them [13]. Furthermore, the study revealed that the higher the levels of enjoyment that a teacher experiences during the course of engaging in his/her profession, the lower the amount of emotional labor he/she would need to exert [13]. Finally, the emotion of anger is likely to cause an increase in the amount of emotional labor that a teacher engages in, which is in turn, a correlate of emotional exhaustion—one of the primary causes of burnout and its attendant symptoms [13].

In a study of 703 Chinese teachers, the researchers observed that the way teachers perceive the school climate may increase exertions spent on emotional labor, which in turn could cause emotional exhaustion at work [14]. Furthermore, emotional labor expended on surface acting was significantly correlated with emotional exhaustion among Chinese teachers, while there were no significant relationships between emotional labor spent on deep acting and emotional exhaustion [14]. The study [14] also reported that while emotional labor spent on deep acting may lead to some degree of burnout, it may also lead to an increase in the all-round wellbeing of teachers.

### 3.2. Impact of emotional labor on the health of hotel workers

Lee, Moon, Lee, and Kim (2014) [15] carried out a survey of 1320 hotel employees working in 5 Seoul hotels and discovered that there was a high correlation between emotional labor and fatigue, as well as emotional dissonance, which has been proven to have harmful effects on the psychological wellbeing of employees. The study also revealed that female hotel workers were likelier engage in emotional labor, consequently leading to higher levels of fatigue than their male counterparts [15]. Fatigue, as a result of excessive emotional, physical and mental labor, can disrupt an individual's state of homeostasis and result in both physical and psychological illnesses [15].

### 3.3. Impact of emotional labor on the health of nurses

Zamanzadeh, Valizadeh, Sayadi, Taleghani, Howard, and Jeddian (2013) [16] conducted a qualitative study of 18 Iraninan female nurses and argues that since women are usually more emotionally engaged than members of the opposite sex, they are likelier to be more engaged in emotional labor. The study also reported that the efforts expended by nurses in carrying out emotional labor during the course of their profession could cause them personal emotional pain, which may lead to emotional exhaustion and depression [16].

### 3.4. Impact of emotional labor on sales and call center employees

In a study of 975 Seoul women who worked in sales and call centers, researchers discovered that emotional labor was significantly associated with dysmenorrhea [17]. This could be attributed to the fact that emotional labor can induce stress, which elicits hormonal imbalances that can ultimately lead to irregularities in the menstrual cycle [17].

Chung, Jung, Kim, and Cho (2017) [17], did a cross-sectional study of 583 female Korean sales personnel working in a clothing shopping mall. The results of their study revealed that since these employees were exposed to numerous occupational stressors, they had to expend emotional labor in coping with them [18]. The study also revealed that emotional labor was one of the key factors that increased the chances of developing depression in sales workers [18].

### 3.5. Impact of emotional labor on the health of dental hygienists

In a study of 807 female Korean dental hygienists, the investigators observed that the absence of a protective/supportive system in the organization led to an increase in emotional labor, which in turn could lead to burnout [19]. Another study also inferred that emotional labor spent in suppressing emotions at the workplace could eventually result in disruptions in sleep patterns, as well as suicidal tendencies which are rooted in depression and anxiety problems [19].

### 3.6. Impact of emotional labor on toll collectors

Joo and Rhie (2017) [20] studied 264 female Korean toll collectors to examine their emotional labor and workplace violence status. In their discussion, the researchers were able to infer that the toll collectors were liable to suffer from depression as a result of the high intensity of emotional labor during the course of discharging their duties [20]. Furthermore, in their discussion, the researchers inferred that some of the benefits of engaging in emotional labor include better blood circulation as a result of smiling, as well as a general sense of wellbeing due to the release of certain neurotransmitters when emotional labor is done [20]. On the negative side, the researchers discussed that emotional labor may lead to the onset of neural problems, musculoskeletal malfunctions, subjective dysesthesia and cardiovascular diseases [20].

### 3.7. Impact of emotional labor on researchers

In this qualitative paper, McGarrol (2017) [21] reveals how researching into the health issues of 50 heart attack survivors (39 men and 11 women) left her emotionally exhausted, particularly due to the emotional labor expended in the course of carrying out the research. Consequently, emotional exhaustion could have undesirable impacts on the psychological health and wellbeing of the researchers [21].

### 3.8. Impact of emotional labor on bank employees

A Korean study of 278 bank employees revealed that a subset of emotional labor—referred to as “emotional disharmony”—was related to increased chances of the manifestation of depressive symptoms [1]. The study also reported that organized surveillance of employees facilitated an increase in the emotional labor that the bank employees needed to expend in carrying out their activities. This increase in emotional labor, further led to an increase in emotional disharmony, as well as emotional exhaustion which is positively correlated with depression [1].

## 4. Conclusion

This paper presents a narrative review of literature showcasing the effects of emotional labor on the health of members of the population who belong to the working class. The very fact that emotional labor can have an impact on the health of individuals is a classic case of reciprocal determinism, as explained in Glanz, Rimer and Viswanath's (2008) [22] explanations of the Social Cognitive Theory (SCT):

SCT emphasizes reciprocal determinism in the interaction between people and their environments. Most behavioral and social theories focus on individual, social, and environmental factors that determine individual or group behavior (for example, barriers, rewards and punishments, and social norms portrayed in mass communication). SCT posits that human behavior is the product of the dynamic interplay of personal, behavioral, and environmental influences. Although it recognizes how environments shape behavior, this theory focuses on people's potential abilities to alter and construct environments to suit purposes they devise for themselves. In addition to a person's individual capacity to interact with their environment, SCT emphasizes the human capacity for collective action. This enables individuals to work together in organizations and social systems to achieve environmental changes that benefit the entire group. (p. 170).

In all the papers that were reviewed, influences from the environment in the form of clients, supervisors, or organizational culture put employees in a position where they had to develop the habit of engaging emotional labor to cope with physical and mental stresses facilitated by these groups of people. As summarized in Table 1, a high number of employees experience health conditions which affects the quality of their work produced and could gradually develop into lifelong ailments. As shown in this paper, this can have deleterious effects on the health of the workforce, which could further translate into loss in productivity as hours that could have been spent on working, is spent on sick leaves. The result of this is a disruption in workers' careers which could lead to them being laid off their jobs. Further research shows that these factors can be reduced through trainings of employee on emotional management, conducive work environment and right attitudes towards all employee [23]. Actions taken to reduce emotional labor will have a positive impact regardless of the job intensity.

**Table 1.** Impact of emotional labor on different professions.

Profession	Impact of Emotional Labor
Teachers	Anger and Anxiety
Hotel Workers	Fatigue and Dissonance
Nurses	Muscoskeletal problem, Exhaustion and Depression
Sales and Call center Employee	Dysmenorrhea and Depression
Dental Hygienists	Disruption in sleep patterns, Suicidal Tendency. Anxiety and Depression
Toll Collectors	Muscoskeletal Malfunction, Subjective Dysesthesia, Cardiovascular Diseases and Depresson
Researchers	Psychological Imbalance
Bank Employer	Emotional Disharmony, Depression and Exhaustion

## 5. Highlights

There is an increase in the level of importance ascribed to the delivery of services as more economies are making the leap from a manufacturing-oriented economy to one that is service-oriented. This leads to the expenditure of emotional labor which has been found to be associated with effects ranging from burnout and fatigue to dysmenorrhea, disruptions in sleep patterns and suicidal tendencies. Emotional labor can occur in any type of profession, some of which causes long term effects on a person's health.
